# A soluble starch synthase I gene, *IbSSI*, alters the content, composition, granule size and structure of starch in transgenic sweet potato

**DOI:** 10.1038/s41598-017-02481-x

**Published:** 2017-05-24

**Authors:** Yannan Wang, Yan Li, Huan Zhang, Hong Zhai, Qingchang Liu, Shaozhen He

**Affiliations:** Key Laboratory of Sweet potato Biology and Biotechnology, Ministry of Agriculture/Beijing Key Laboratory of Crop Genetic Improvement/Laboratory of Crop Heterosis and Utilization, Ministry of Education, China Agricultural University, Beijing, 100193 China

## Abstract

Soluble starch synthase I (SSI) is a key enzyme in the biosynthesis of plant amylopectin. In this study, the gene named *IbSSI*, was cloned from sweet potato, an important starch crop. A high expression level of *IbSSI* was detected in the leaves and storage roots of the sweet potato. Its overexpression significantly increased the content and granule size of starch and the proportion of amylopectin by up-regulating starch biosynthetic genes in the transgenic plants compared with wild-type plants (WT) and RNA interference plants. The frequency of chains with degree of polymerization (DP) 5–8 decreased in the amylopectin fraction of starch, whereas the proportion of chains with DP 9–25 increased in the *IbSSI*-overexpressing plants compared with WT plants. Further analysis demonstrated that IbSSI was responsible for the synthesis of chains with DP ranging from 9 to 17, which represents a different chain length spectrum *in vivo* from its counterparts in rice and wheat. These findings suggest that the *IbSSI* gene plays important roles in determining the content, composition, granule size and structure of starch in sweet potato. This gene may be utilized to improve the content and quality of starch in sweet potato and other plants.

## Introduction

In plants, starch consists of amylose and amylopectin. Amylose mainly comprises linear chains that are linked by α-1, 4 O-glycosidic bonds, whereas amylopectin is highly branched and contains 5–6% α-1,6 O-glycosidic bonds to generate glucan branches of various lengths^1^. The biosynthesis of starch in plants is mainly mediated by four classes of enzymes: ADP-glucose pyrophosphorylase (AGPase), starch synthase, starch branching enzyme (SBE) and starch debranching enzyme (DBE)^2^.

To date, great progress has been achieved in understanding the physicochemical properties of different types of starch synthases and their functions in glucan chain elongation and starch granule formation. Starch synthase can be grouped into five types, granule-bound starch synthase (GBSS) and four types of soluble starch synthases (SS): SSI, SSII, SSIII and SSIV. In cereals, GBSSI is encoded by the *Waxy* (*wx*) locus and functions mainly to synthesize amylose^3^, but it also contributes to the synthesis of long amylopectin chains^4^. The loss of GBSSI function does not significantly alter the starch content of rice^5^, maize^6^ or wheat^7, 8^, but these mutants show reduced amylose content or a complete lack of amylose. In contrast, SSI, SSII and SSIII are mainly involved in amylopectin synthesis by mediating chain elongation. In rice, three genes encode SSII (*SSII*-*1*, *2*, *3*), two genes encode SSIII (*SSIII*-*1*, *2*), SSIV (SS*IV*-*1*, *2*) and GBSS (*GBSSI*, *II*), and only one gene encodes SSI^9^. The loss of SSII decreases the proportion of amylopectin chains with DP 11–30 and increases the accumulation of amylose^10–14^. The mutation of rice *SSIIIa* reduces the content of long chains (DP ≥ 30) in the endosperm and results in smaller and rounder starch granules and floury-like endosperm, without affecting the seed starch content^15, 16^. Deficiency in maize SSIII decreased the proportion of DP 36 to 53 chains^17^. These results indicate that the elongation of long glucan chains is mainly due to SSIII and not SSII. The number of starch granules per plastid is decreased and the granule size is increased in *Arabidopsis* mutants that carry defective *SSIV*, which suggests that this gene is involved in the initiation stages of starch granule biosynthesis rather than in the elongation of amylopectin chains^18^. Unlike the other three types of soluble starch synthases (SSII, SSIII and SSIV), which have been found to contain multiple isoforms, SSI has no known isoform^19^. It is suggested that SSI may play a specific role in starch biosynthesis.

In rice, evidence has shown that both *in vivo* and *in vitro* SSI mainly synthesizes DP 8–12 chains by adding glucose residues to the short B1 and B2 chains that emerge from the branching points or to the A chains with DP 6–7. Meanwhile, a deficiency in rice SSI does not significantly impact the amylose content or morphology of rice endosperm starch granules, which suggests that other types of SS may compensate for the partial function of SSI^2^. Two mutants generated by T-DNA insertion into the *Arabidopsis SSI* gene demonstrated that AtSSI was most active on glycogen with an average outer chain length (OCL) of 7–8 compared with maize amylopectin (OCL = 12–14) and maize β-limit dextrin (OCL = 2.5). This length is consistent with the preferable DP value of 6–7 that is often modified by rice SSI. Meanwhile, the mutant accumulates less starch than WT *Arabidopsis* ^1^. Similarly to AtSSI, maize SSI is most active for glycogen substrates with an OCL ranging from 7 to 9^20^.

Sweet potato, *Ipomoea batatas* (L.) Lam., is an important root crop with a high yield per hectare. It is a mainstay of food consumption in several undeveloped regions because it is a source of high dietary energy for humans and can resist abiotic and biotic stresses^21^. In addition to its contribution to human subsistence, the starchy root of this crop, which can be processed into bio-ethanol, is widely used in the energy industry^22^. Nevertheless, improving the content and quality of sweet potato starch remains an urgent demand, especially in the field of biotechnology.

In sweet potato, several genes that encode the starch biosynthetic enzymes have been cloned and characterized^13, 14, 23–27^. These genes have been reported to impact the starch composition, structure and physicochemical properties and minimally influence the starch content. However, the role of SSI in starch biosynthesis of sweet potato has not been reported. In this study, we isolated the soluble starch synthase I gene (*IbSSI*) from the sweet potato. The sweet potato was genetically manipulated to overexpress or suppress *IbSSI*, which affected the content, composition, structure and physicochemical properties of starch. These findings will contribute to the improvement of starch content and quality in sweet potato for use in the biofuel industry.

## Results

### Isolation and sequence analysis of *IbSSI*

**Figure 1:**
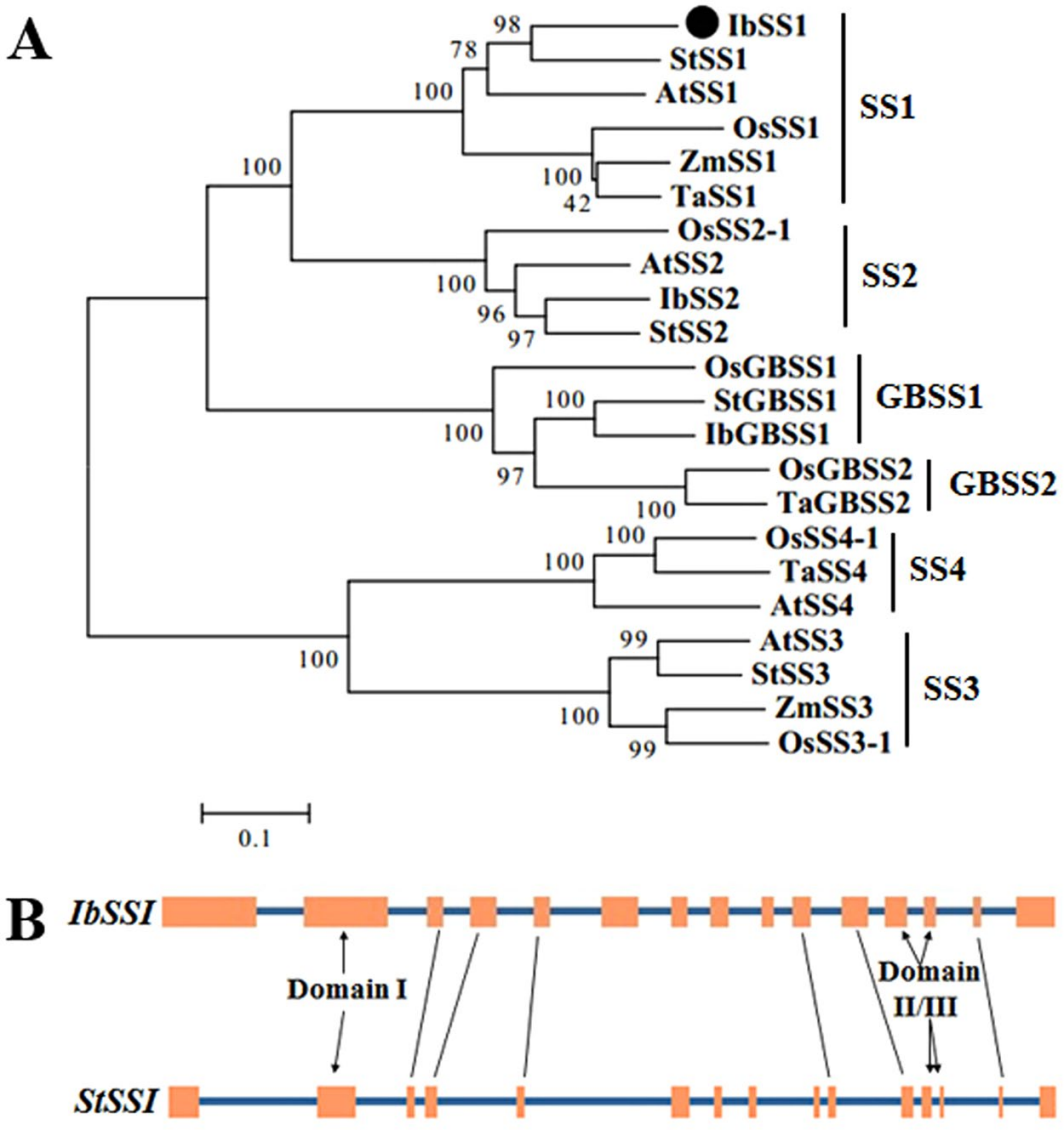
Sequence analysis of *IbSSI*. (**A**) Phylogenetic tree of the starch synthases of different plant species generated using MEGA 6.0 with the neighbor-joining method. The *IbSSI* cloned in this study is indicated by a black spot. The complete species names of the starch synthases and their GenBank accession numbers are listed in Supplementary Table S2. (**B**) Comparison of the genomic structures of *IbSSI* (*Ipomoea batatas*) and *StSSI* (*Solanum tuberosum*). The orange boxes represent the exons and the blue lines indicate the introns. Distributions of the three conserved domains are illustrated by black arrows.

The *IbSSI* gene was cloned from a high-starch sweet potato line Xu 781 using RACE methods. The cloned 2440-bp full-length *IbSSI* cDNA contained a 1974-bp ORF that produced a polypeptide with a molecular weight of 72.9 kDa and a predicted pI of 5.34. A search for the amino acid sequence of IbSSI in NCBI returned a putative glycogen synthase domain that featured an ADP-binding pocket (Supplementary Fig. S1). A multiple alignment of IbSSI with the SSI from potato, *Arabidopsis*, maize and wheat using DNAMAN revealed three domains that were highly conserved in the plant starch synthases and *E. coli* glycogen synthases^28, 29^ (Supplementary Fig. S2). Among the three domains, domain I contained a K-*x*-G-G-L motif which is included in the ADP-glucose binding motif ^30^. The binding of ADP-glucose to IbSSI is a key step in elongating the glucan polymers. A phylogenetic analysis further revealed (Fig. 1A) that IbSSI is more closely related to the potato (*Solanum tuberosum*) StSSI, and the proteins that clustered together with IbSSI all belonged to the soluble starch synthase I family. The genomic sequence of *IbSSI* was 4027 bp in length. An analysis of the genomic structure using the Spidey program revealed that *IbSSI* contained 15 exons and 14 introns, and this structure is highly similar to that of potato *StSSI*. Furthermore, domains I, II and III in *IbSSI* and *StSSI* were distributed in the same exons, with domain I in the second exon, domain II in the twelfth exon and domain III spanning the twelfth and thirteenth exons (Fig. 1B). However, the genomic structures of *IbSSI* and *StSSI* significantly differed. Each exon of *IbSSI* is longer than the corresponding *StSSI* exon, but the total length of the introns in *StSSI* (6480 bp) is three times the length of introns in *IbSSI* (2053 bp). These findings suggest that *IbSSI* may contain less regulatory sequences than *StSSI*, which may be responsible for their differences in expression patterns and biological functions. The 5′-promoter region (~1800 bp) of *IbSSI* was isolated and a number of *cis*-acting regulatory elements were detected by PlantCARE (Supplementary Table S4).

### IbSSI activity in *E. coli*

The cloned *IbSSI* gene was expressed in *E. coli Trans*etta (DE3) to examine its starch synthase activity. An SDS-PAGE of the cell lysate that contained the IbSSI recombinant protein indicated that *IbSSI* could be successfully expressed in the prokaryotic system (Supplementary Fig. S3). The specific activity of soluble starch synthase from *E. coli* cells that contained the recombinant IbSSI (29.50 units/mg protein) increased 3.86-fold compared with those that contained the native pET-28a vector (6.07 units/mg protein) (Supplementary Table S3). This result confirmed that *IbSSI* encodes a functional starch synthase.

### Expression analysis of *IbSSI* in sweet potato

**Figure 2:**
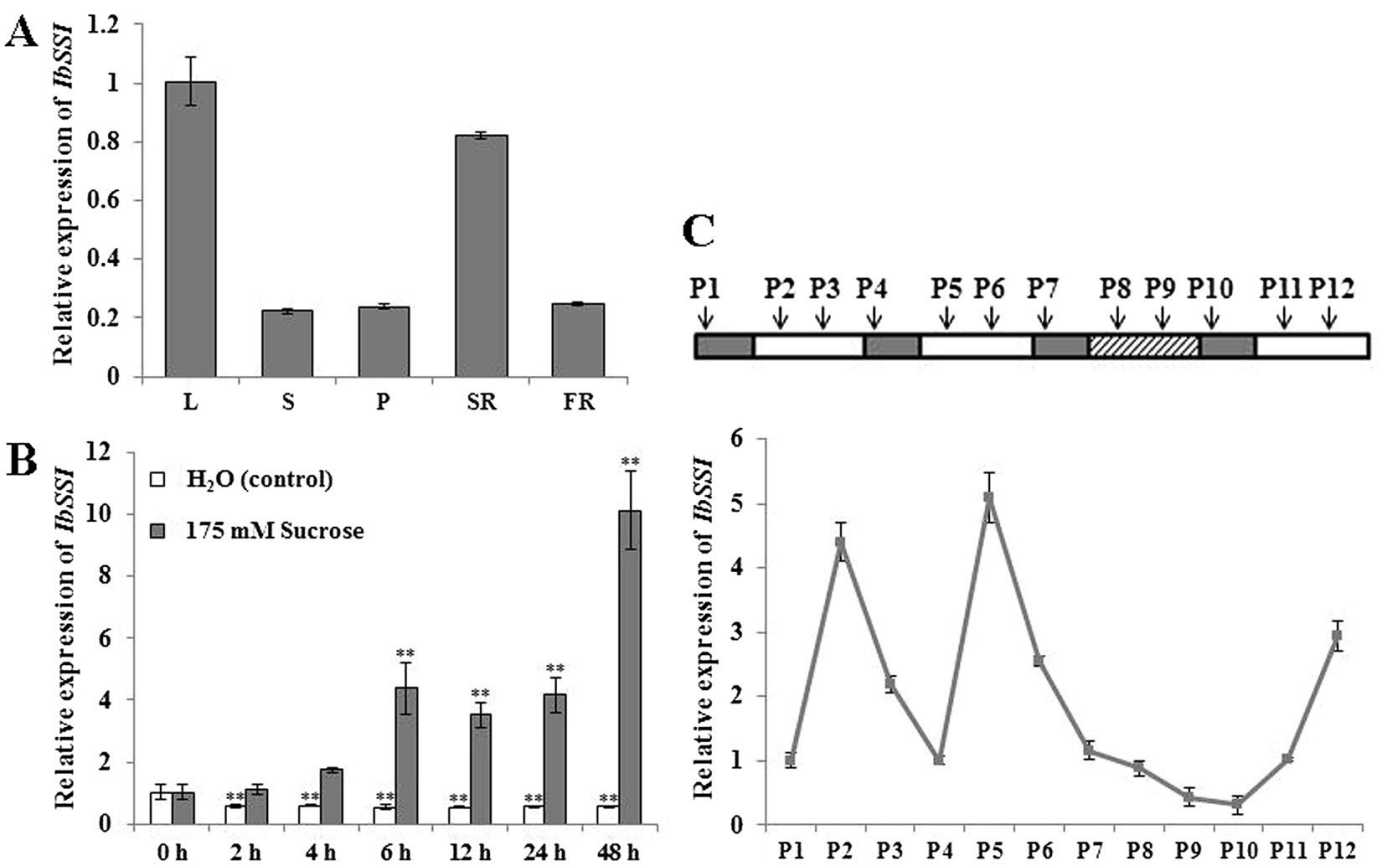
Expression analysis of *IbSSI* in the sweet potato. (**A**) Transcript levels of *IbSSI* in different tissues of the sweet potato detected by qRT-PCR. L, leaf; S, stem; P, petiole; SR, storage root; FR, fibrous root. (**B**) Expression profile of *IbSSI* in response to 175 mM sucrose treatment. (**C**) Time course of *IbSSI* expression detected by qRT-PCR during 16 h light/8 h dark photoperiods with light on at 5 am and off at 9 pm. Total RNA was extracted from the *in vitro*-grown whole plant sampled at 10 pm (P1, P4, P7 and P10), 10 am (P2, P5, P8 and P11) and 4 pm (P3, P6, P9 and P12), respectively. The open bars represent the 16 h light period, the solid bars correspond to the 8 h dark period, and the hatched bars indicate the extended dark period that would otherwise be the light period. The results are expressed as relative values with respect to the L, 0 h and P1, which were set to 1.0 in (**A–C**), respectively. Data are presented as the mean ± SD (n = 3). **Indicates a significant difference versus the control at *P* < 0.01 based on Student’s *t*-test.

The spatial expression pattern of *IbSSI* was investigated by qRT-PCR. The results revealed that *IbSSI* was expressed in all five major tissues of Xu 781, with much higher levels detected in the leaves and storage roots than in petioles, stems and fibrous roots (Fig. 2A). This expression pattern is similar to that of another starch biosynthetic gene in sweet potato, *IbSBEII* ^31^.

To examine whether exogenous sucrose treatment could induce the expression of *IbSSI*, leaf-petioles of Xu 781 were treated with either water (control) or 175 mM sucrose for 48 h. Treatment with water did not up-regulate *IbSSI* expression in leaf-petioles (Fig. 2B). In contrast, exogenous sucrose treatment could significantly enhance the expression of *IbSSI*. After 48 h of treatment, the transcript level of *IbSSI* showed 9-fold up-regulation in response to the sucrose treatment.

The circadian clock of plants is endogenous and sustained in a cycling manner with a free-running period of approximately 24 h^32^. A time-course experiment was designed to determine whether expression of *IbSSI* follows the circadian rhythm (Fig. 2C). The level of *IbSSI* mRNA was high at 10 am (P2, P5) in the first two normal 24 h photoperiods and showed severe reduction during the 32 h extended dark period (P7-P10). This result indicated that the light supply was the external clue that sustained the rhythmic oscillation of *IbSSI* mRNA level in the normal 24 h photoperiod and the circadian clock does not regulate the expression of *IbSSI*. The level of *IbSSI* mRNA began to restore when the external light was resumed (P11, P12).

### Subcellular localization of IbSSI protein

**Figure 3:**
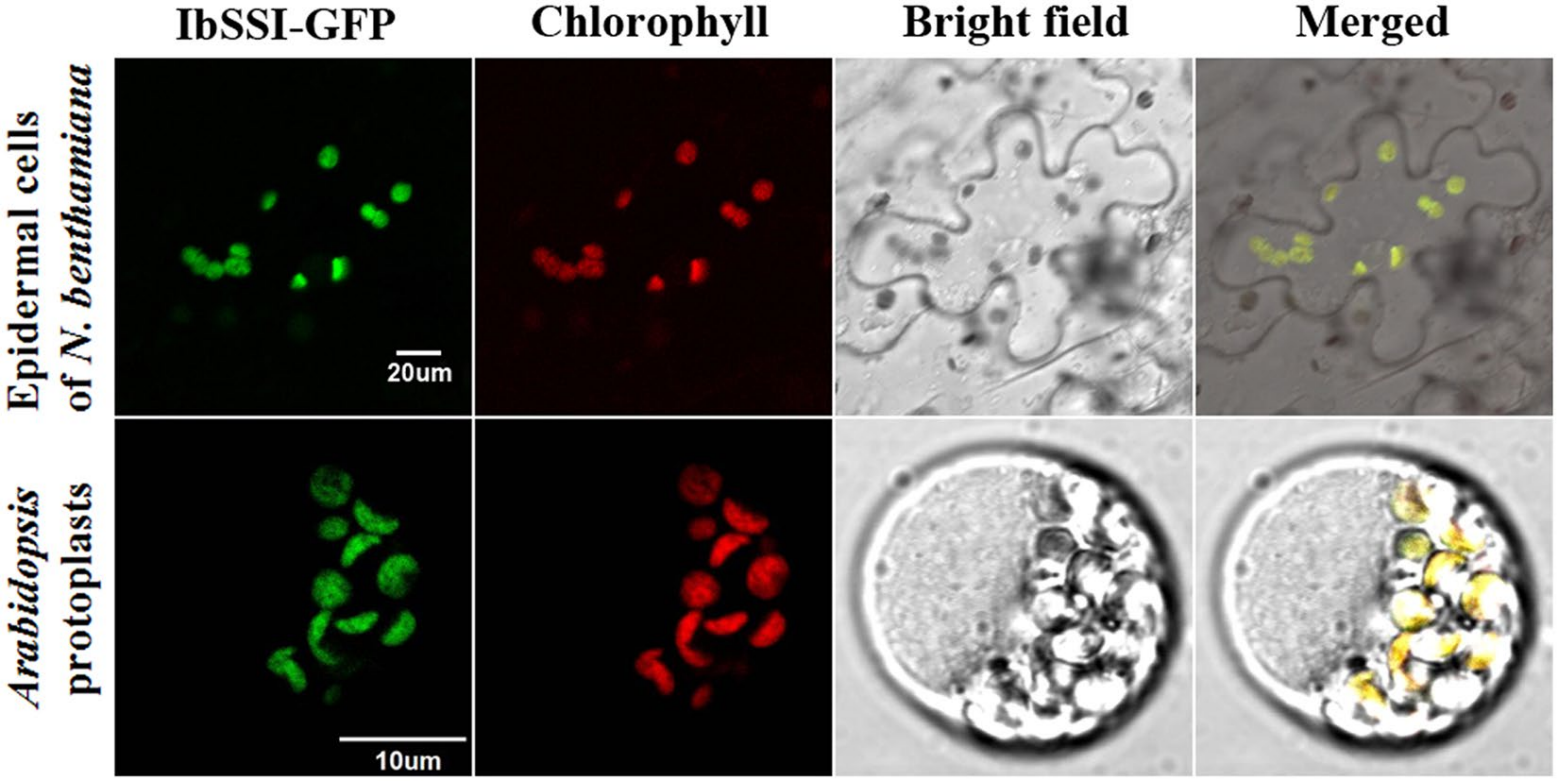
Subcellular localization of IbSSI protein. Upper row: confocal images of green fluorescence of IbSSI-GFP fusion protein are shown in *N. benthamiana* leaf hypodermal cells, which co-localized with the autofluorescence of chloroplasts. Scale bar = 20 μm. Lower row: co-localization of IbSSI-GFP signals with autofluorescence of chloroplasts in *Arabidopsis* mesophyll protoplast. Scale bar = 10 μm.

The subcellular localization of IbSSI protein was examined in *N. benthamiana* epidermal cells and *Arabidopsis* protoplasts. As shown in Fig. 3, in *N. benthamiana* epidermal cells, IbSSI was observed as scattered patches and co-localized with the autofluorescence of chloroplasts. In *Arabidopsis* protoplast, signals associated with the IbSSI were also localized to the chloroplasts. Furthermore, signals of IbSSI were observed to spread throughout the stroma of chloroplasts both in *N. benthamiana* epidermal cells and *Arabidopsis* protoplasts, which is similar to the localization pattern of AtSSI in *N. benthamiana* chloroplasts^33^. These results indicated that IbSSI is localized to chloroplasts, wherein starch biosynthesis occurs.

### Production of transgenic sweet potato plants

To functionally characterize *IbSSI*, we constructed its overexpression vector (pC3301–121-*IbSSI*) and RNAi vector (pFGC5941-*IbSSI*) (Supplementary Fig. S4) and introduced them into the low-starch sweet potato cultivar Lizixiang, respectively. A total of 217 putative transgenic plants that overexpressed *IbSSI* were generated (denoted L1, L2, …, L217), and a GUS staining assay and PCR analysis confirmed that *IbSSI* was overexpressed in 34 of these plants. Furthermore, 125 putative RNAi plants (denoted Li1, Li2, …, Li125) were obtained, and PCR analysis indicated that *IbSSI* was successfully knocked down in only 12 of these plants. These 12 RNAi plants (RNAi lines) and another 12 *IbSSI*-overexpressing plants (overexpression lines) that were randomly selected from the 34 aforementioned plants were planted in the soil in the greenhouse and then in the field. Storage roots were harvested after 5 months of growth in the field. Production of transgenic sweet potato plants is illustrated in Supplementary Fig. S5.

**Figure 4:**
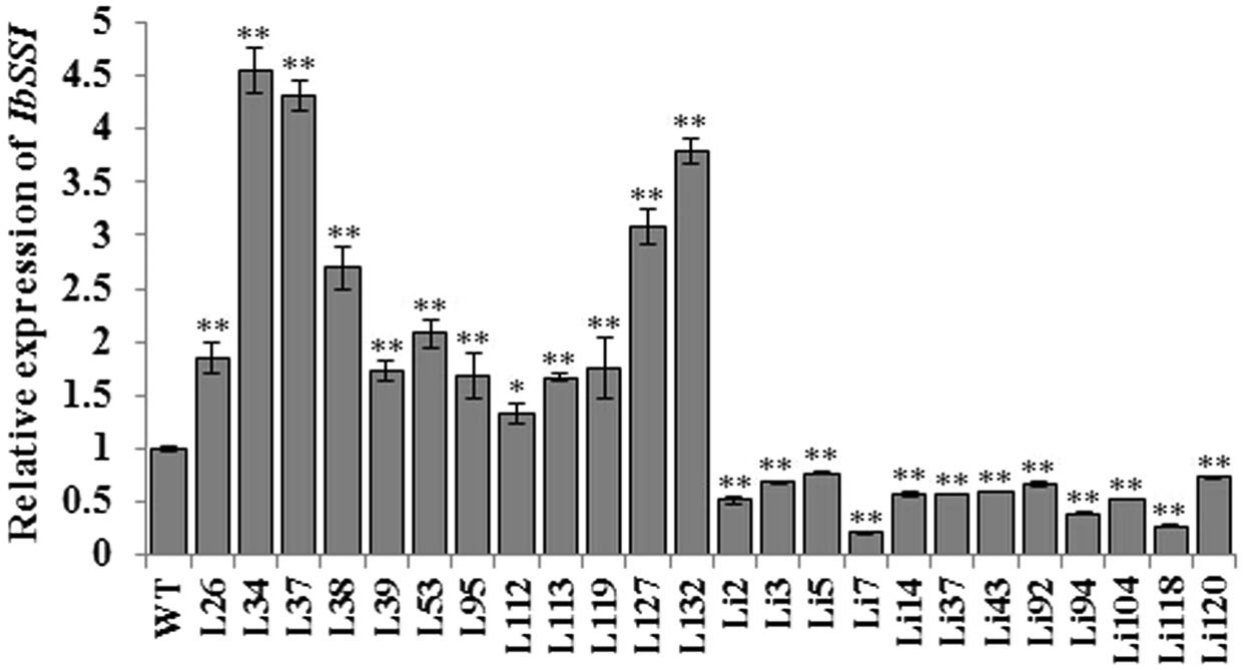
Transcript levels of *IbSSI* in the storage roots of the 24 transgenic lines and WT. The results are expressed as relative values with respect to WT, which was set to 1.0. Data are presented as the mean ± SD (n = 3). *And **Indicate a significant difference versus the WT at *P* < 0.05 and <0.01, respectively, based on Student’s *t*-test.

The transcript level of *IbSSI* in the storage roots of the 24 transgenic lines and WT was investigated by qRT-PCR (Fig. 4). Compared with WT, the transcript level of *IbSSI* was highest in three overexpression lines (L34, L37 and L132) and lowest in two RNAi lines (Li7 and Li118). These five lines were selected for further analyses.

### Amounts of starch-granule-bound proteins

**Figure 5:**
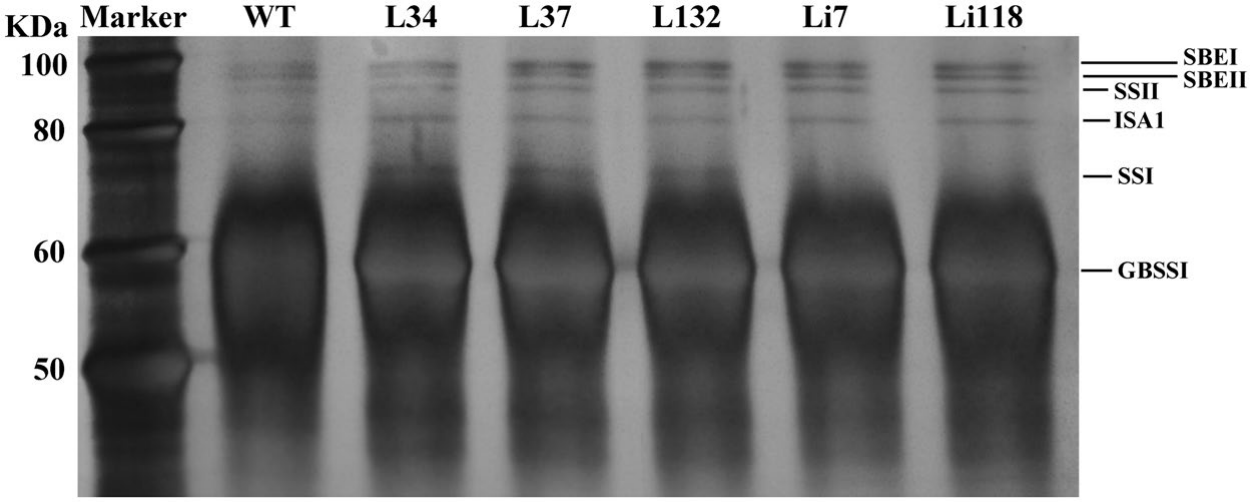
Detection of starch-granule-bound proteins in the transgenic lines and WT. A total of 5 mg of purified starch from each line were boiled in 80 μL SDS buffer for 5 min and centrifuged at 15,000 g. Equal amount of supernatant (25 μL) were loaded into the gel. The proteins were visualized by silver staining.

Starch-granule-bound proteins were extracted from 30 mg of starch granules and separated by SDS-PAGE and detected by silver staining (Fig. 5). The major granule-bound proteins detected include GBSSI, SSI, ISA1, SSII, SBEI and SBEII. In WT starch, slight quantity of SSI was detected. The amount of SSI protein increased in the overexpression lines, whereas the two RNAi lines showed no detectable abundance of SSI protein. The other granule-bound proteins remained unchanged in the transgenic lines.

### Starch content and composition

The starch and amylose contents were quantified in the storage roots of the five transgenic lines and WT (Table 1). The starch content was significantly increased in two of the overexpression lines (L37 and L132) and significantly reduced in lines Li7 and Li118 compared with WT. To characterize the starch composition, we used the colorimetric method and Con A method to measure the amylose content. The overexpression of *IbSSI* significantly reduced the the relative proportion of amylose compared with WT when examined by both methods (Table 1) and the colorimetric measurement was about 4.6–10.2% higher than the Con A method. In contrast, the relative proportion of amylose was increased in the RNAi lines. These results demonstrate that *IbSSI* can impact the content and composition of starch in sweet potato.

**Table 1:** Starch content and composition of the transgenic plants and WT^a^.

Lines	Starch content	Relative proportion of amylose (%)	
	(mg g^−1^ FW)	Colorimetric method	Con A method
WT	120.5b (3.0)	19.29b (0.23)	18.45c (0.44)
L34	122.1b (3.5)	17.44c (0.18)	16.67e (0.22)
L37	148.9a (1.7)	17.19c (0.38)	15.60 f (0.43)
L132	153.3a (2.7)	18.95b (0.14)	17.29d (0.28)
Li7	103.4c (3.0)	21.29a (0.23)	20.37b (0.30)
Li118	108.3c (2.6)	21.68a (0.14)	21.03a (0.14)

^a^Data are presented as the mean of three replicates (starch samples from three different storage roots) with standard deviation given in the parenthesis. The values in the same column with different letters differ significantly at *P* < 0.05, based on Student’s *t*-test.

### Starch granule size and morphology

**Figure 6:**
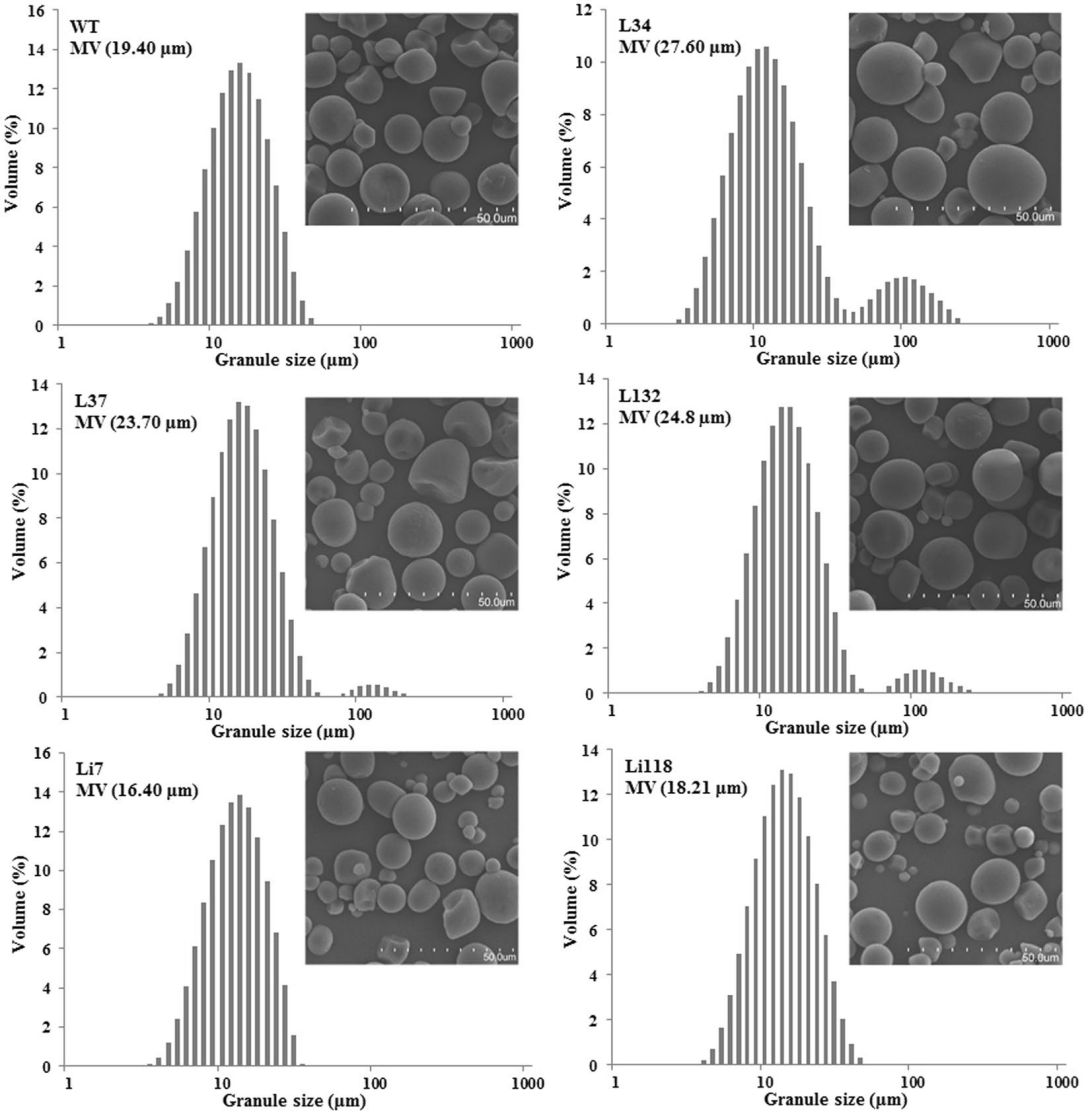
Size distribution and mean volume diameter (MV) of starch granules from the transgenic plants and WT. Insets: Scanning electron micrographs of the starch granules from the transgenic plants and WT. The dotted line indicates a length of 50.0 µm.

As shown in Fig. 6, the starch granule size distributions in the transgenic lines differed from that of WT. Unlike the unimodal patterns observed in the RNAi lines and WT, two peaks appeared in the overexpression lines at approximately 13 μm and 104 μm. The overexpression lines exhibited a broader granule size distribution than WT and contained a greater number of larger starch granules. This part of larger starch granules was largely responsible for the increased average granule size (MV value) in the overexpression lines. In contrast, the average granule size of the RNAi lines was smaller than that of WT because the RNAi lines exhibited a narrower granule size distribution (Li7, size distribution: 2.93–37.66 μm) or a smaller proportion of larger granules (Li118) than WT (size distribution: 3.33–55.24 μm).

The starch granules that had been purified from the transgenic plants and WT were examined by SEM (Fig. 6, insets). The granules from the overexpression lines were slightly larger than those from WT, whereas the RNAi lines contained a greater number of smaller granules. No other significant morphological alterations were observed in the five lines. Each line contained multiple granular shapes, but the characteristic oval shape was maintained, and no fissures were observed in the granules. These morphological characteristics were similar to those observed by Noda *et al*.^34^ in sweet potato. Some incomplete-spherical and polygonal granules were detected in each sample, which were likely due to immature development or physical damage during the granule purification process.

### Chain length distribution (CLD)

**Figure 7:**
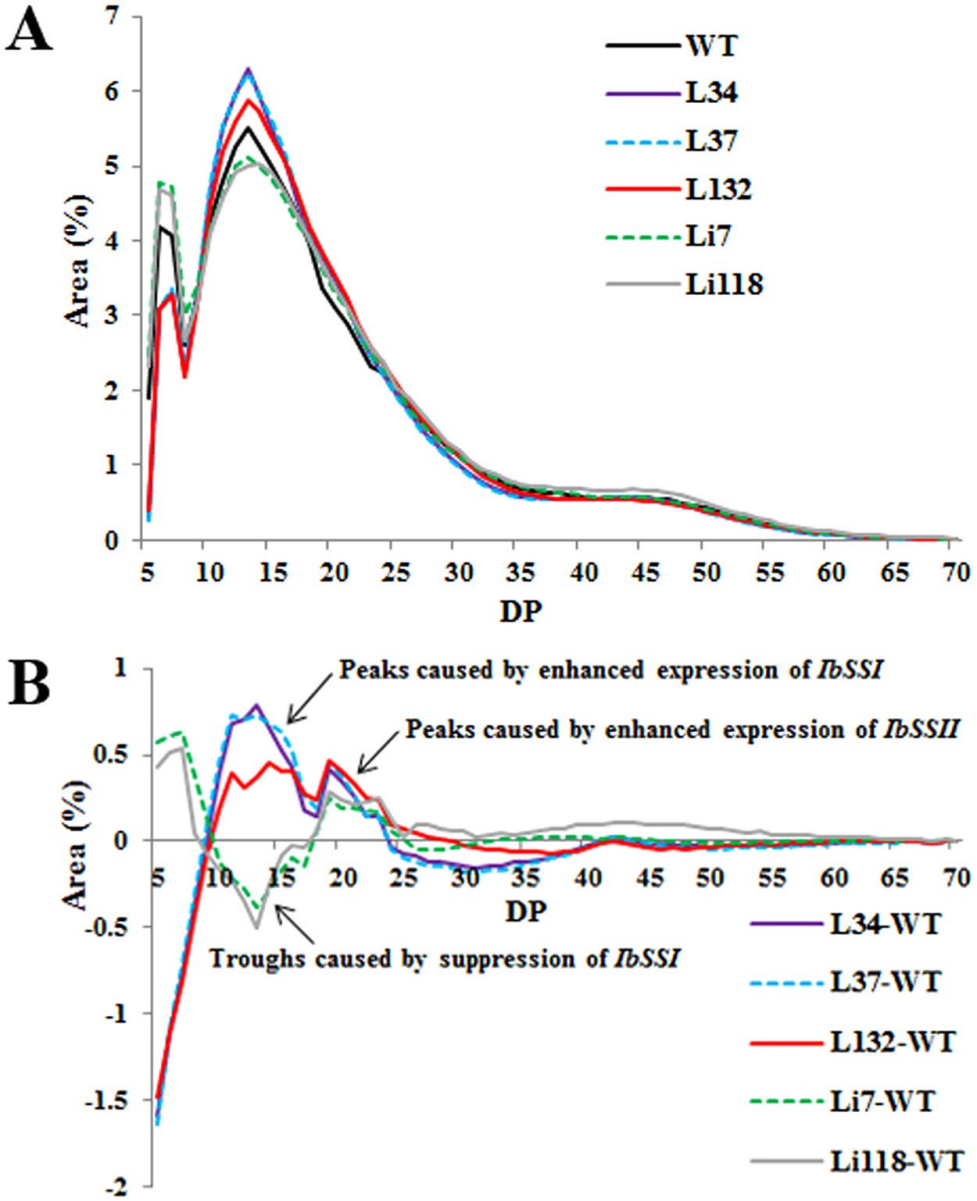
Amylopectin chain length distributions of the transgenic plants and WT. (**A**) CLD of the starch isolated from the five transgenic lines and WT after normalization to the total peak area. (**B**) Differences in the CLD between the transgenic lines and WT were calculated as follow: the normalized CLD value for each transgenic line minus the value obtained for the WT.

The amylopectin CLD of DP 5–70 in the transgenic plants and WT were calculated based on the peak areas (Fig. 7A). The CLD pattern of each line was similar to that of WT. A trough at DP 8 and two peaks at around DP 14 and DP 47 were observed. This pattern is consistent with previous findings^22^. The value obtained for the amylopectin glucan chain of WT was subtracted from the corresponding value for the transgenic lines to generate the CLD difference model (Fig. 7B). In the overexpression lines, the number of chains with DP 5–8 and DP 26–40 decreased, while the number of chains with DP 9–25 increased. Between DP 9–25, the magnitude of the change peaked at approximately DP 14 and then decreased to a trough until another peak appeared at DP 19. The change in the CLD observed for Li7 and Li118 was almost a mirror image of those observed for L34, L37 and L132 between DP 5–18, which displayed an increase for chains with DP 5–8 and DP ≥ 18 and a decrease for chains with DP 9–17. These CLD patterns were different from those in sweet potatoes, in which *IbSSII* was inhibited^13, 14^. The opposing CLD alteration patterns between the overexpression and RNAi lines indicated that the *IbSSI* gene might play a key role in the synthesis of amylopectin chains with DP 9–17 (short to intermediate) in sweet potato.

### X-ray diffraction measurement

**Figure 8:**
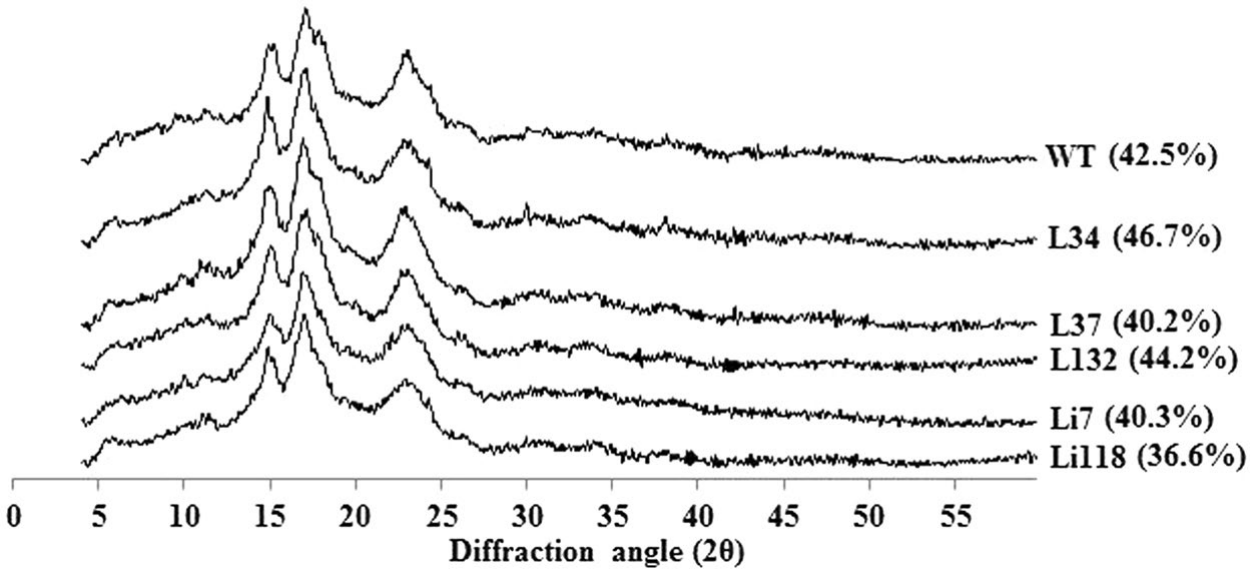
Wide-angle X-ray powder diffraction spectra for starches from WT and different transgenic lines. All the starch samples showed typical A-type crystal pattern. The percentage in the parentheses are the degree of crystallinity.

The X-ray diffractograms of the starch samples from different transgenic lines and WT are presented in Fig. 8 and the degree of crystallinity are shown in parentheses. All the starch samples exhibited A-type crystal patterns with strong reflections at 2*θ* of about 15° and 23°. An unresolved double peak at 2*θ* of 17° and 18° were also found. This result was similar with the observation in rice, in which the mutation of *SSI* did not change the A-type crystal pattern of the control line^2^. In WT starch, approximately 43% crystallinity was detected whereas L34 and L132 showed higher crystallinity. However, reduction of crystallinity was observed in Li7 and Li118. The changes of crystallinity of starch from the transgenic lines were probably caused by the variations in their relative proportion of amylose as revealed by Cheetham and Tao^35^. Even so, the changes in amylose content or starch CLD were not big enough to alter the crystal patterns of the starch from the transgenic lines.

### Thermal analysis of starch

The DSC parameters of the starch samples were determined in order to evaluate the gelatinization properties (Table 2). The starch of WT gelatinized with a temperature range of 61.9 °C (*T*_o_) to 81.5 °C (*T*_c_) and an enthalpy (∆*H*) of 12.1 J g^−1^. The gelatinization temperatures (*T*_o_, *T*_p_ and *T*_c_) were decreased in the five transgenic lines and showed clear differences in comparison with WT. This tendency is different from that observed by Fujita *et al*.^2^ and McMaugh *et al*.^36^ in rice and wheat. In their studies, suppression of *SSI* gene led to 1 °C to 3 °C higher gelatinization temperatures. Compared with WT, L34 starch had significantly higher ∆*H* while Li7 starch showed lower ∆*H* during gelatinization. No significant differences in ∆*H* were demonstrated for starch from other transgenic lines.

**Table 2:** DSC analysis of starch from the transgenic plants and WT^a^.

Lines	*T*_o_ (°C)	*T*_p_ (°C)	*T*_c_ (°C)	∆*H* (J g^−1^)
WT	61.9a (0.1)	72.1a (0.1)	81.5a (0.5)	12.1b (0.8)
L34	58.8b (0.1)	63.6d (0.2)	81.7a (0.5)	15.4a (0.7)
L37	58.6b (0.2)	68.8c (0.1)	79.6b (0.6)	12.3b (0.6)
L132	57.7c (0.2)	70.2b (0.7)	80.8a (0.1)	12.1b (1.3)
Li7	57.7c (0.1)	61.3 f (0.3)	69.7d (1.0)	9.0c (0.8)
Li118	58.0c (0.1)	62.9e (0.2)	73.5c (0.6)	11.1b (0.5)

^a^Data are presented as the mean of three replicates with standard deviation given in the parenthesis. The values in the same column with different letters differ significantly at *P* < 0.05, based on Student’s *t*-test.

### Pasting properties of starch

The pasting properties of starch were analyzed by the RVA and the results are presented in Table 3. The pasting temperatures of the five transgenic lines were lower than that of WT. The peak viscosity (PV) values were lower in the overexpression lines and higher in the RNAi lines than in WT. The breakdown (BD) value did not differ between the five lines and WT. And the values for hot paste viscosity (HV), final viscosity (FV) and setback (SB) were significantly lower in the overexpression lines than in WT, while no typical pattern was identified for the RNAi lines.

**Table 3:** RVA analysis of starch from the transgenic plants and WT^a^.

Lines	Peak viscosity (cP)	Hot paste viscosity (cP)	Breakdown (cP)	Final viscosity (cP)	Setback (cP)	Peak time (min)	Pasting temperature (°C)
WT	3331.7c (7.6)	1895.0b (18.0)	1463.3c (22.5)	2619.7a (29.1)	724.7bc (19.7)	10.2c (0.0)	70.7a (0.4)
L34	3207.0d (12.2)	1684.3c (29.8)	1522.7b (26.0)	2418.0b (22.5)	733.7b (8.0)	10.1c (0.0)	70.0a (0.1)
L37	2809.7e (23.7)	1315.7d (23.8)	1494.0b (47.5)	1962.7c (25.5)	647.0d (18.4)	9.9d (0.2)	67.8b (0.6)
L132	2685.3 f (36.9)	1293.3d (17.9)	1392.0c (19.0)	1874.7d (24.5)	581.3e (7.37)	9.8d (0.0)	66.9c (0.4)
Li7	3489.7b (11.5)	1961.0a (3.5)	1528.7b (12.1)	2650.3a (27.0)	689.3c (23.7)	10.5b (0.0)	66.1d (0.5)
Li118	3928.3a (29.7)	1855.3b (32.9)	2073.0a (20.9)	2625.0a (23.1)	769.7a (31.1)	10.7a (0.0)	65.1e (0.5)

^a^Data are presented as the mean of three replicates with standard deviation given in the parenthesis. The values in the same column with different letters differ significantly at *P* < 0.05, based on Student’s *t*-test.

### Expression of starch biosynthetic genes and enzyme activity assay

**Figure 9:**
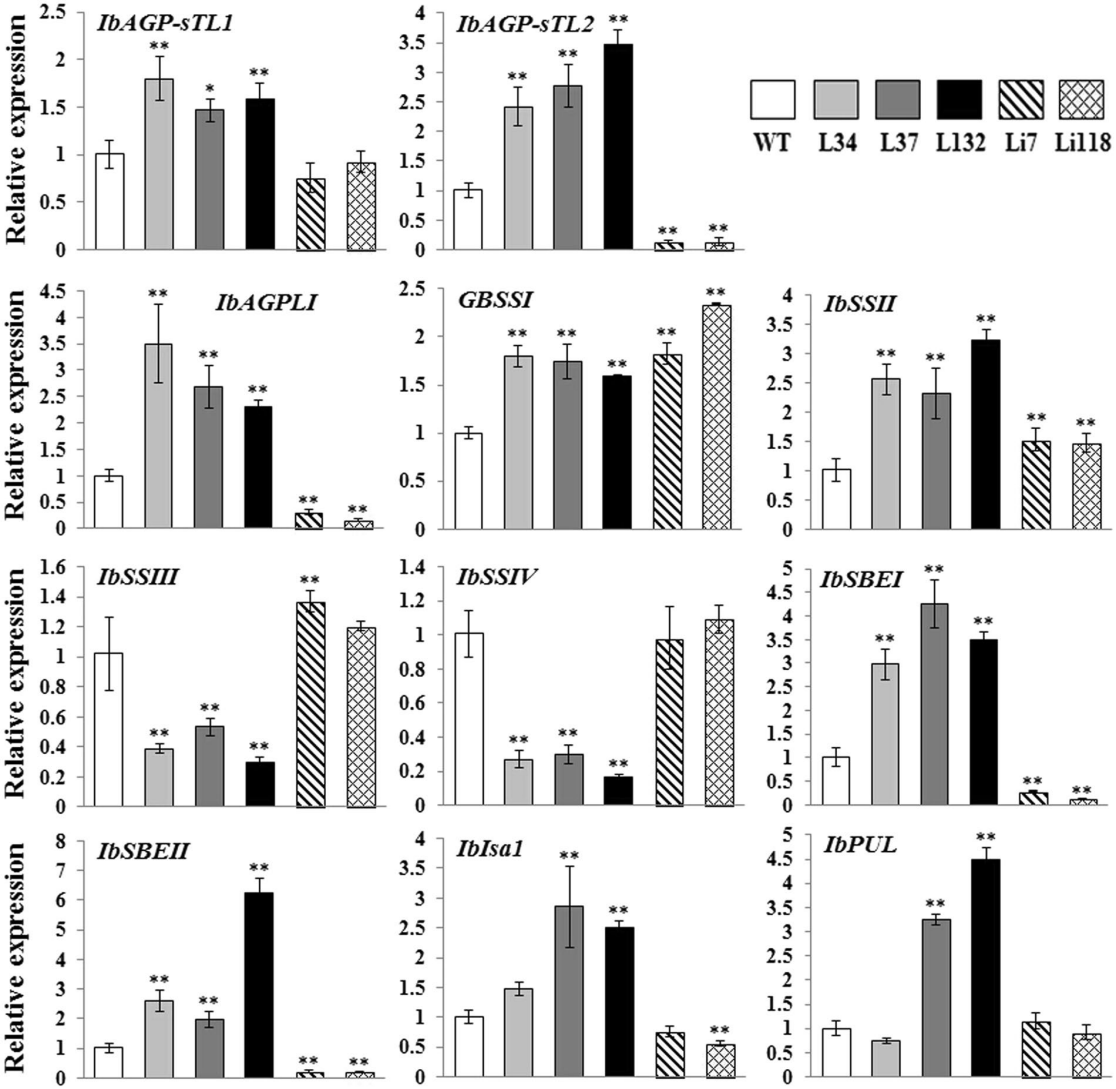
Transcript levels of 11 starch biosynthetic genes in the storage roots of the transgenic plants and WT. The results are expressed as relative values with respect to WT, which was set to 1.0. Data are presented as the means ± SD (n = 3). *And **Indicate a significant difference versus WT at *P* < 0.05 and <0.01, respectively, based on Student’s *t*-test.

The transcript levels of 11 starch biosynthetic genes in the five transgenic lines were examined by qRT-PCR (Fig. 9). Six of the 11 genes were up-regulated in the overexpression lines and down-regulated in the RNAi lines (*IbAGP-sTL1*, *IbAGP-sTL2*, *IbAGPLI*, *IbSBEI*, *IbSBEII*, *IbIsa1*). In contrast, *IbSSIII* and *IbSSIV* showed lower transcript levels in the overexpression lines and higher transcript levels in the RNAi lines, while the transcript levels of *IbGBSSI* and *IbSSII* were increased in all the five transgenic lines. The *IbPUL* gene was significantly up-regulated in two of the overexpression lines (L37, L132) and showed no significant difference in transcript levels in lines L34, Li7 and Li118 compared with WT.

**Figure 10:**
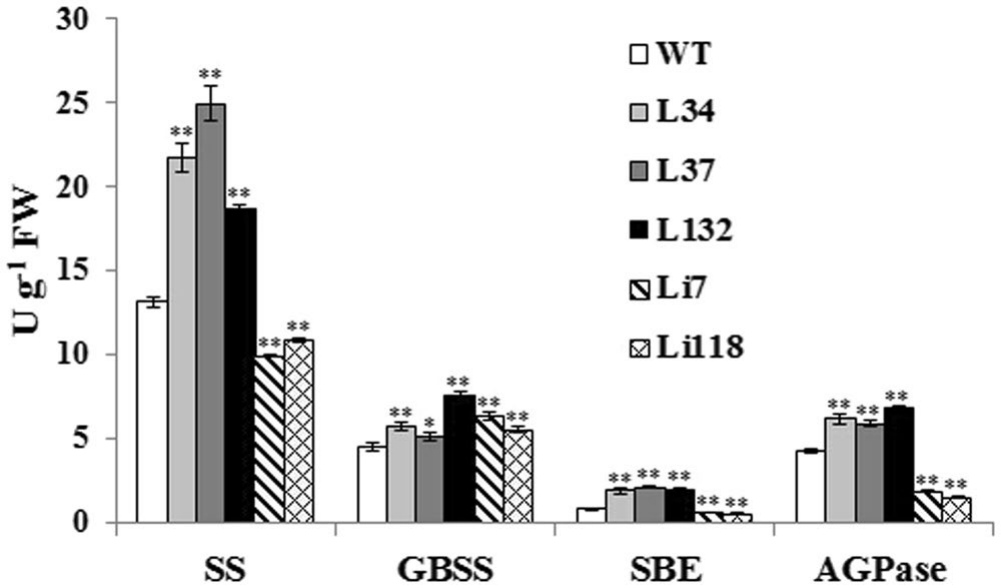
Enzyme activities of SS, GBSS, AGPase, SBE in the storage roots of the transgenic plants and WT. Data are presented as the mean ± SD (n = 3). *And **Indicate a significant difference versus WT at *P* < 0.05 and <0.01, respectively, based on Student’ s *t*-test.

The enzyme activity levels of the four starch biosynthetic enzymes (SS, GBSS, AGPase and SBE) depended on the transgenic lines (Fig. 10). The activity of SS was significantly higher in the overexpression lines and lower in the RNAi lines compared with WT. The activity of another starch synthase, GBSS, was enhanced in all transgenic lines. The activities of both SBE and AGPase were increased in the overexpression lines and reduced in the RNAi lines compared with WT. The changes in the activity of each enzyme in transgenic plants were basically consistent with the alterations in the transcript levels of their corresponding genes.

## Discussion

In sweet potato, the spatial expression pattern of *IbSSI* showed similarity to that of *IbSBEII* and major difference from that of other starch biosynthetic genes from sweet potato, such as *IbAGP-sTL1*, *IbAGP-sTL2*, *IbGBSSI* and *IbSBEI* ^23, 24, 31, 37^. In potato, the mRNA level of *StSSI* is drastically lower in tubers than that in sink and source leaves, which might suggest a minor role of StSSI in starch biosynthesis in the storage organs^38^. However, the present study indicated that the transcript level of *IbSSI* was much higher in leaves and storage roots than in petioles, stems and fibrous roots (Fig. 2A). This suggests that *IbSSI* plays active roles in the starch biosynthesis in both leaves and storage roots.

Sucrose is an important regulatory signal involved in starch metabolism^39^. Starch synthesis is induced when the supply of carbohydrates (either endogenous or exogenous) exceeds the respiratory demand^40^. In the present study, pre-treatment with water in the dark depleted the endogenous carbohydrate and reduced the respiration to a low level. Consequently, floating the leaf-petiole in sucrose triggered starch synthesis and induced *IbSSI* gene expression. A similar trend was observed for the sweet potato genes *IbSpo*, *IbGBSSI* and *IbAGP-sTL1* ^23, 41, 42^. Furthermore, a sucrose-responsive *cis*-acting regulatory element (Carbohydrate Metabolite Signal Responsive Element 1, CMSRE 1, TGGACGG) was detected at the -379 position of the 5′-promoter region of *IbSSI* (Supplementary Table S4). This element has been shown to be the core motif that determines the response of several other genes to sucrose^43–45^ and may also play a role in the response of *IbSSI* to sucrose treatment. Both experimental and sequence analyses collectively indicated that *IbSSI* is sucrose inducible.

Starch and amylose content are important agronomic traits for root crops. Many studies have been conducted to investigate the regulatory effects of *SS* genes on these two traits^10, 15, 16, 18, 38, 46^. The *SS* genes in these studies were down regulated by RNAi or mutated by T-DNA insertion, which showed no clear rules of alterations in starch and amylose contents. This lack of rule was attributed to the following. Firstly, *SS* are responsible for the synthesis of amylopectin and the down-regulation of either type of *SS* may affect the amylose/amylopectin ratio without modifying the total starch content, provided that the total carbon influx does not change. Secondly, other type of *SS* could functionally compensate for the down-regulation or mutation of one specific gene (for example, overlapping functions of *SSII* and *SSIII* in *Arabidopsis* ^12^). Thirdly, environmental factors may play a role. In the present study, the overexpression and suppression of *IbSSI* oppositely affected the starch and amylose content. As shown in Figs 9 and 10, rather than being suppressed as a result of the overexpression of *IbSSI*, the expression level of *IbGBSSI* and its enzymatic activity were increased in lines L34, L37 and L132, which likely facilitated the synthesis of amylose. This phenomenon probably occurred because of siphon-like regulation: the overexpression of *IbSSI* increased the consumption of carbon resources for the synthesis of amylopectin, and this over-exhaustion may have increased the carbon flux into the starch biosynthetic pathway, which up-regulated the expression of *IbAGP* and *IbGBSSI*. However, the increase in expression of *IbGBSSI* was smaller than the overall increase in expression of *IbSSI* and *IbSSII* (Figs 9 and 10), and the unbalanced increase in *IbGBSSI* and *IbSSI* (and *IbSSII*) expression decreased the relative proportion of amylose. Additionally, the up-regulation of the transcript level and enzymatic activity of *IbSBE* in the overexpression lines also decreased the relative proportion of amylose, as described by Kitahara *et al*.^27^. Moreover, the collective enhancement of the activities of AGPase, GBSS and SS contributed to the accumulation of starch in the storage roots of the overexpression lines. The RNAi lines exhibited different activity patterns in the starch biosynthetic genes. The activity of AGPase was significantly decreased in RNAi lines, which reduced the starch content. The activity of SS in Li7 and Li118 showed significantly but relatively small reduction compared WT, likely due to the enhanced activity of SSIII and/or SSIV. The increase in the GBSS activity and the decrease in the SBE activity may together result in the accumulation of amylose in these two lines.

In the present study, the suppression of *IbSSI* increased the proportion of chains with DP 5–8 and DP ≥ 18 and reduced the proportion of chains with DP 9–17. Thus, we assume that the DP 5–8 chains accumulated because they were not elongated efficiently by IbSSI to DP 9–17 chains, whose proportion decreased subsequently. Meanwhile, the available DP 9–17 chains were further converted to longer chains with DP 18–25 by IbSSII, which was up-regulated in the RNAi lines (Fig. 9). This was similar with the rice *SSI* mutant, in which the deficiency of OsSSI leaves more short chains with DP 8–12 suitable for OsSSII to generate longer chains Fujita *et al*.^2^. Previous study showed that maize SSI displayed an affinity for an average chain length of DP 7–14 and its affinity for chains of DP > 20 is significantly lower because it becomes entrapped within the starch granule^20^. In this study, overexpression of *IbSSI* increased the amount of chains with DP 9–25 and decreased the chains with DP 5–8, with two peaks and a trough appearing at DP 13, DP 19 and DP 17, respectively (Fig. 7B). Accordingly, it is suggested that in sweet potato, DP 18 represents the time at which IbSSI became inactive. Based on this assumption, chains with DP 5–8 in the overexpression lines decreased because the elevated levels of IbSSI converted these chains to additional DP 9–17 chains. Subsequently, these chains were further elongated by IbSSII to DP 18–25 chains. Thus, the up-regulation of *IbSSI* and *IbSSII* in the overexpression lines gave rise to the two peaks at DP 13 and DP 19. Collectively, these results suggest that IbSSI is mainly responsible for the synthesis of amylopectin chains with DP 9–17, which is different from the preferable DP spectrum of SSI from rice (8–12) and wheat (8–12)^2, 36^. Concurrently, the transgenic lines contained some slight alterations in chains with DP 26–55 compared with WT, which could be attributed to the alteration in expression levels of *IbSSIII* and/or *IbSSIV* (Fig. 9), as described in previous studies^16, 47, 48^. Nevertheless, additional evidence is needed to explain the changes in CLD in the transgenic lines based on the mechanism underlying the interaction between the starch biosynthetic genes.

The altered expression of *IbSSI* in the transgenic lines changed the size distribution of the starch granules, possibly due to the significantly differed amylose content. In the *Arabidopsis SSI* and rice *SSIIIa* mutant lines^1, 27^, enrichment of amylose content caused more smaller starch granules, which was similar to lines Li7 and Li118 in the present study. Conversely, the average granule size positively correlated with the amylose content in the overexpression lines (L34, L37 and L132). It is noteworthy that a new fraction of larger granules appeared in the overexpression lines, which may imply an alteration in the starch granule initiation caused by enzymatic changes of SSIII or SSIV as revealed by Szydlowski *et al*.^49^. And this maybe was another reason that the average size of the starch granules was increased. The Morphological analysis of the starch granules in the five lines by SEM revealed no significant differences compared with the WT. The morphological features of starch granules in rice *SSI* mutant lines deficient in *OsSSI* did not significantly differ from those WT rice^2^. In contrast, the starch granules from the potato *SSIII* mutant lines were deeply fissured on the hilum^47^. Therefore, the *IbSSI* gene could impact granule size by influencing the synthesis of amylopectin or amylose. A deficiency in the *IbSSI* did not cause defects in granule morphology, likely because of the overlapping functions provided by other types of soluble starch synthase.

Different types of starch crystalline structures have been found in root and tuber plants. The starch from cassava exhibits the A-type crystallinity^50^, whereas potato and yam are the B-type^51, 52^. Both A-type and C-type were reported in different cultivars of sweet potato^22, 53–55^. In the present study, the crystal structure of starches in transgenic lines and WT was A-type. The present results further indicated that the peak viscosity values were higher in the RNAi lines and lower in the overexpression lines than in WT. This result is inconsistent with those of previous studies. The PV of the rice *SSI* mutant line was 77% of that of the control line^2^. Many studies of crops that produce waxy starches, such as waxy wheat and waxy barley^56, 57^, have demonstrated that a reduction of the amylose content increased the PV value of starch. However, the PV value of waxy potato was lower than that of normal potato starch^58^, and this was similar to the decreased PV values observed in the overexpression lines in this study. Meanwhile, the lower PV values in the overexpression lines may also be due to their starch containing more intermediate chains (DP 13–25) and less short chains (DP 5–8), which was similar to the lower PV value of the wheat *SSI* suppression line^36^. No typical patterns were identified for HV, FV and SB values in the RNAi lines, which was likely due to the pleiotropic regulation of *IbSSI* on other starch metabolic genes, or some other factors were involved such as lipid content of the starch.

In conclusion, the *IbSSI* gene has been successfully isolated from a high starch sweet potato line Xu 781. Its overexpression significantly increased the starch content and granule size, and decreased the amylose/amylopectin ratio in transgenic sweet potato plants. This gene is also responsible for the synthesis of short to intermediate chains with DP 9–17. These results suggest that *IbSSI* has great potential to be used to improve the content and quality of starch in sweet potatoes and other plants.

## Methods

### Plant materials

The sweet potato line Xu 781 with high starch content was employed for the isolation of the *IbSSI* gene. A low-starch sweet potato cultivar Lizixiang was used to characterize the function of this gene through determining if its starch could be improved or not. Both Xu 781 and Lizixiang were cultivated in a greenhouse under a regimen of 16 h light/8 h dark at 28 °C.

### Isolation of *IbSSI* cDNA, genomic DNA sequences and 5′-promoter region

Total RNA was extracted from the leaves of Xu 781 and transcribed into first-strand cDNA with the PrimeScript II 1^st^ Strand cDNA Synthesis Kit (TaKaRa, Dalian, China). A pair of degenerate primers (DS-F/R) was designed based on the most homologous region identified by aligning the SSI sequence from different plant species. An EST fragment was amplified from the first-strand cDNA. Subsequently, rapid amplification of cDNA ends (RACE) was conducted to obtain the full-length cDNA with gene-specific primers (GSPs). The genomic sequence of *IbSSI* was amplified with GS-F/R primers using genomic DNA extracted from the leaves of Xu 781 as a template. The 5′-promoter region of *IbSSI* gene was isolated using the Universal GenomeWalker 2.0 Kit (TaKaRa, Dalian, China). GWS1 and GWS2 were used as gene specific primers. The sequences of the primers used in this study are listed in Supplementary Table S1.

### Sequence analysis of *IbSSI*

The open-reading frame (ORF) of the cloned *IbSSI* gene was predicted using the ORF Finder (http://www.ncbi.nlm.nih.gov/projects/gorf/). The homology of the IbSSI protein was identified using the protein blast in NCBI (http://blast.ncbi.nlm.nih.gov/Blast.cgi). The molecular weight and theoretical isoelectric point (pI) of the IbSSI protein were calculated using http://web.expasy.org/compute_pi/. A multiple sequence alignment of the IbSSI protein with other SSI proteins was conducted using the DNAMAN software. The genomic structure of *IbSSI* was analyzed with the Spidey program (http://www.ncbi.nlm.nih.gov/spidey/). A phylogenic tree was constructed using the MEGA 6.0 software with the neighbor-joining (NJ) method. The *cis*-acting regulatory elements in the 5′-promoter region of *IbSSI* were searched using PlantCARE (http://bioinformatics.psb.ugent.be/webtools/plantcare/html/).

### Assessment of IbSSI activity in *Escherichia coli*

The ORF of *IbSSI* was amplified using the ES-F/R primers and inserted into the pET-28a vector. Next, the sequence-validated pET-28a-*IbSSI* vector and native pET-28a vector were introduced into competent *E. coli* strain *Trans*etta (DE3) cells (Transgen Biotech, Beijing, China). Positive clones were grown in Luria-Bertani (LB) medium. Fresh LB medium was inoculated with the overnight cultures at a 100:1 (v:v) dilution. Soluble cytoplasmic proteins were prepared from isopropyl β-D-thiogalactopyranoside (IPTG)-induced *Trans*etta (DE3) cells^59^. The expressed IbSSI protein was subjected to SDS-PAGE analysis according to the method of Jiang *et al*.^60^, and a starch synthase assay was conducted as described previously^61^. One unit of activity was defined as the formation of 1 nmol of ADP per min at 30 °C.

### Expression analysis of *IbSSI* in the sweet potato

The transcript levels of *IbSSI* were analyzed in five different tissues (storage root, fibrous root, stem, leaf and petiole) of Xu 781 grown for 100 days in a field. Total RNA was extracted from each tissue and reverse transcribed into cDNA and quantitative real-time PCR (qRT-PCR) was conducted using the SYBR detection protocol (TaKaRa) with the ABI 7500 Real-Time PCR system. The primers used to amplify *IbSSI* and *IbActin* (endogenous control) are listed in Supplementary Table S1.

The response of *IbSSI* to exogenous sucrose was investigated as described by Wang *et al*.^42^ with some modifications. Leaf-petioles (10 cm) from Xu 781 grown for 100 days in a field were cultured for 1 day in water in the dark and then treated with either water (control) or 175 mM sucrose in the dark at 28 °C. qRT-PCR was conducted to determine the mRNA levels of *IbSSI* in cuttings harvested at different time points (0, 2, 4, 6, 12, 24 and 48 h) after treatment.

The response of *IbSSI* to changes of light conditions was examined as described by Leterrier *et al*.^62^. Twelve *in vitro*-grown plants of Xu 781 were acclimated to a 16-h-light/8-h-dark regimen in a growth chamber at 28 °C for 1 month prior to modifying the light conditions. Total RNA was extracted from each whole plant sampled at different time points (shown in Fig. 2C) and the mRNA levels of *IbSSI* were subsequently detected by qRT-PCR.

### Subcellular localization of the IbSSI protein

To construct the IbSSI-GFP fusion protein, the ORF of *IbSSI* was amplified from the first-strand cDNA with primers 83S-F/R. The sequence-validated ORF fragment was then ligated into the pMDC83 vector. This recombinant vector was introduced into the *Agrobacterium tumefaciens* strain EHA105 and used for transient expression in *Nicotiana benthamiana* leaf epidermal cells according to the method of Strasser *et al*.^63^. Meanwhile, *Arabidopsis* mesophyll protoplasts were also used for the subcellular localization of IbSSI. Protoplast isolation and vector transfection were conducted as described previously^64^. After co-cultivation, the agroinfiltrated tobacco leaves and transfected *Arabidopsis* protoplasts were visualized with a laser scanning confocal microscope (Nikon Inc., Melville, NY, USA).

### Production of transgenic sweet potato plants

The ORF of *IbSSI* was amplified using OS-F/R primers and then inserted into pBI121 between the *Xba*I and *Sac*I sites to replace the glucuronidase (*gusA*) gene. Next, the expression cassette *35S-IbSSI-NOS* was excised from the pBI121-*IbSSI* vector with *Hind*III and *EcoR*I and then ligated between the same cleavage sites in pCAMBIA3301 to generate the overexpression vector. To construct the RNA interference (RNAi) plasmid, two fragments (FDF and RDF) were amplified from the coding sequence of *IbSSI* using the primers Si-UF/R and Si-DF/R and inserted into the cleavage sites of *Xho*I/*Swa*I and *BamH*I/*Xba*I in the pFGC5941 vector, respectively. The sequence of the recombinant vector pFGC5941-*IbSSI* was validated. The two recombinant plasmids for overexpression and RNAi were transfected into *A. tumefaciens* strain EHA105, respectively. Embryogenic suspension cultures of Lizixiang used for Agrobacterium infection were established with the method of Liu *et al*.^65^. The procedures for embryogenic transformation, selection of resistant calluses and plant regeneration were as described by Liu *et al*.^66^ except that phosphinothricin and cefotaxime sodium were added in the selection culture.

The putatively transgenic sweet potato plants overexpressing *IbSSI* were identified using the histochemical GUS assay according to Jefferson *et al*.^67^. The blue staining of tissues indicated a positive reaction. Genomic DNA was extracted from the leaves of these GUS-positive plants, and PCR amplifications were performed using the primers T35-F and TS-R to further confirm that these plants were transgenic. To identify the *IbSSI* RNAi plants, genomic DNA was extracted from the leaves of plantlets regenerated from the calluses, and PCR was conducted using the primers int-F/R.

### Expression of starch biosynthetic genes

The transcript levels of *IbSSI* and 11 starch biosynthetic genes in the storage roots of the transgenic plants and wild-type plants (WT) were investigated by qRT-PCR. The starch biosynthetic genes included the following: *IbAGP-sTL1* and *2* (encoding the two small subunits of IbAGPase, respectively), *IbAGP-TLI* (encoding a large subunit of IbAGPase), *IbGBSSI*, *IbSSII*, *IbSSIII*, *IbSSIV*, *IbSBEI*, *IbSBEII*, *IbIsa1* and *IbPUL*. The primers used to amplify these genes are listed in Supplementary Table S1.

### Analysis of starch-granule-bound proteins

Starch-granule-bound proteins were isolated and separated by SDS-PAGE as described previously^68^. After electrophoresis, the proteins were visualized using the Pierce Silver Stain Kit (Thermo Fisher Scientific).

### Enzyme activity assay in storage roots

The activity of four starch biosynthetic enzymes (SS, GBSS, AGPase and SBE) in the storage roots of the transgenic plants and WT was evaluated as described by Nakamura *et al*.^69^. One unit of activity (SS, GBSS and AGPase) was defined as the formation of 1 nmol ADP per min at 30 °C and 1 unit of SBE activity was defined as the amount of enzyme required to increase the spectrophotometric absorbance by 1 unit in 1 minute.

### Quantification of starch content and composition

The starch content in the storage roots of WT and transgenic plants was analyzed according to the method of Smith and Zeeman^70^. Starch was isolated from the sweet potato storage roots as described by Zhao *et al*.^50^. Fresh slices of sweet potato tuberous roots were suspended in distilled water and homogenized in a blender, and the slurry was filtered through a 100-μm sieve. The supernatant was discarded after the sedimentation of the starch granules. This process was repeated three times, and the starch samples were dried in a convection oven at 40 °C for 2 days. The amylose was then quantified using the colorimetric amylose content assay^71^. Standard curves were established with the standard samples of potato amylose and amylopectin (Sigma). Meanwhile, the Concanavalin A (Con A) method was also used to determine the relative proportion of amylose using an amylose/amylopectin assay kit (Megazyme International Ireland).

### Analysis of starch granule size and morphology

The granule size distribution of starch from storage root was determined as described by Zhou *et al*.^22^. A Master-size 2000 laser diffraction instrument (Malvern Instruments Ltd., Worcestershire, UK) was used in wet-well mode. The starch was added to the reservoir and sonicated for 30 s at 6 W until an obscuration value of 12–17% was achieved. The refractive indices used for the water and starch were 1.330 and 1.50, respectively. The particle size distributions are presented as the diameter versus volume^72^. To examine whether the starch granule morphology was altered in different transgenic lines, the starch granules were coated with gold after they were spread on a metal stub and then observed under a scanning electron microscope (SEM).

### Analysis of the chain length distribution (CLD)

The CLD was measured according to Zhou *et al*.^22^. Starch from the transgenic plants and WT was digested with *Pseudomonas amyloderamosa* isoamylase (Sigma), and the CLD of amylopectin was analyzed using high-performance anion-exchange chromatography with pulsed amperometric detection (HPAEC-PAD; Dionex-ICS 3000; Dionex Corporation, USA).

### Analysis of starch granule crystallinity

A D8 Advance Bruker X-ray diffractometer (Bruker AXS, Germany) was used to study the crystal type of the starch granules. Starch powders were scanned with the 2*θ* diffraction angle at 4–50°. The crystal patterns and degree of crystallinity were analyzed using Jade 5.0 software.

### Analysis of thermal characteristics

The thermal properties of the starch samples were examined using a differential scanning calorimeter (DSC Q2000; TA Instrument Ltd., UK). A mixture of starch and distilled water (w/w, 1:3) were sealed in an aluminum pan and scanned at 10 °C min^−1^ from 30 °C to 95 °C with an empty aluminum pan as a reference. The gelatinization temperature and enthalpy were calculated using the Universal Analysis 2000 software.

### Measurement of pasting properties

The pasting properties of the starch were analyzed using a rapid viscosity analyzer (model RVA-Super 3; Newport Scientific Pty. Ltd., Australia). The starch was suspended in distilled water (10% w/v, dry weight basis, 25 mL) and tested using the integrated sweet potato program. The temperature of the starch slurry was increased from 30 °C to 95 °C at a rate of 5 °C min^−1^ and held at 95 °C for 6 min, followed by cooling to 50 °C at the same rate and maintenance for 10 min. The rotating speed of the paddle remained constant (160 rpm) throughout the analysis, excluding the speed of 960 rpm applied during the first 10 s.

## Electronic supplementary material

Supplementary Information
