# Supplementary material for: A soluble starch synthase I gene, IbSSI, alters the content, composition, granule size and structure of starch in transgenic sweet potato
Source: Sci Rep. 2017 May 24;7:2315. doi: 10.1038/s41598-017-02481-x (PMC5443758; doi:10.1038/s41598-017-02481-x)
Supplement: Supplementary file 1 — Supplementary Information [file 41598_2017_2481_MOESM1_ESM.pdf]

A soluble starch synthase I gene, *IbSSI*, alters the content, composition, granule size and structure of starch in transgenic sweet potato

Yannan Wang†, Yan Li†, Huan Zhang, Hong Zhai, Qingchang Liu* & Shaozhen He*

[1]Key Laboratory of Sweet potato Biology and Biotechnology, Ministry of Agriculture/Beijing Key Laboratory of Crop Genetic Improvement/Laboratory of Crop Heterosis and Utilization, Ministry of Education, China Agricultural University, Beijing 100193, China

†These authors contributed equally to this work.

*Correspondence and requests for materials should be addressed to S.H. (email: sunnynba@cau.edu.cn) or Q.L. (email: liuqc@cau.edu.cn)

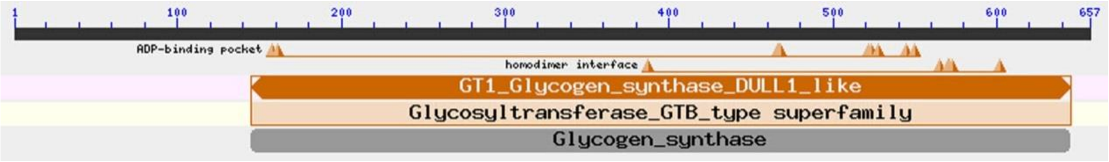

Supplementary Fig. S1. Putative conserved domains detected by a Blast analysis of the IbSSI protein sequence in NCBI. The *IbSSI* gene contains an ADP-binding pocket and a glycogen synthase domain.

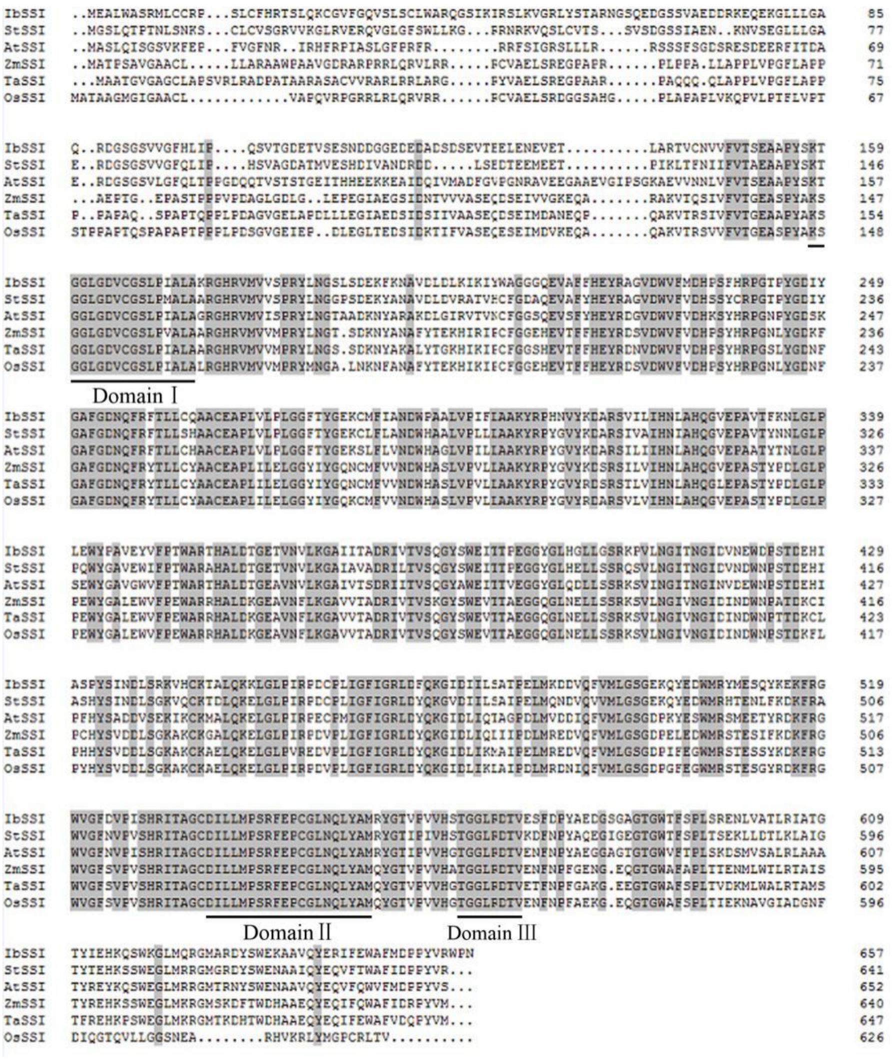

Supplementary Fig. S2. Multiple alignment of the deduced amino acid sequences of starch synthase I from *Ipomoea batatas* (IbSSI), *Solanum tuberosum* (StSSI), *Arabidopsis thaliana* (AtSSI), *Zea mays* (ZmSSI), *Triticum aestivum* (TaSSI) and *Oryza sativa* (OsSSI). Identical amino acids were indicated in the grey background. The three highly conserved domains (I, II and III) of plant starch synthase are highlighted by black lines. The GenBank accession numbers of these soluble starch synthases are listed in Supplementary Table S2.

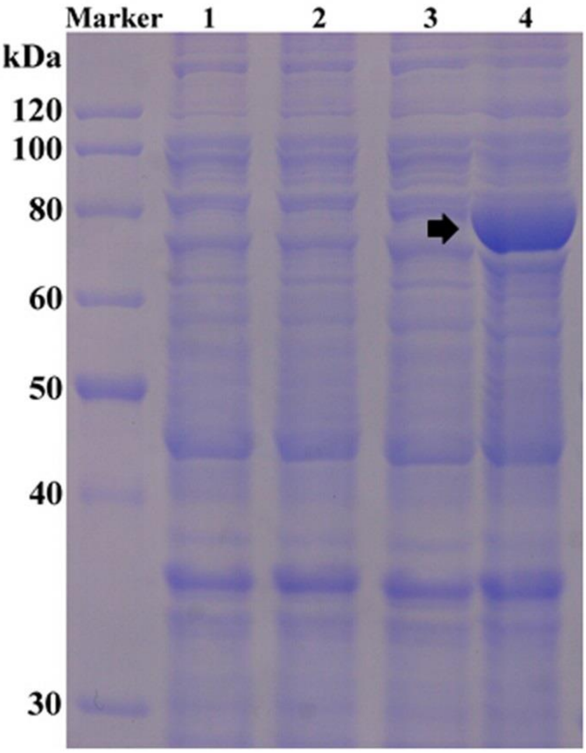

Supplementary Fig. S3. SDS-PAGE of IbSSI protein expressed in bacteria. Lanes 1 and 3, cell lysate from *E. coli Trans*etta (DE3) transformed with the native plasmid pET-28a and pET-28a-*IbSSI* prior to the addition of IPTG. Lanes 2 and 4, cell lysate from *E. coli Trans*etta (DE3) transformed with native plasmid pET-28a and pET-28a-*IbSSI* after induction with 1mM IPTG at 28°C overnight. A band representing the recombinant IbSSI protein was detected at the position of the predicted 72.9 kDa in lane 4 (indicated by an arrow). Protein markers are indicated on the left. The gel was stained with Coomassie Brilliant Blue R-250.

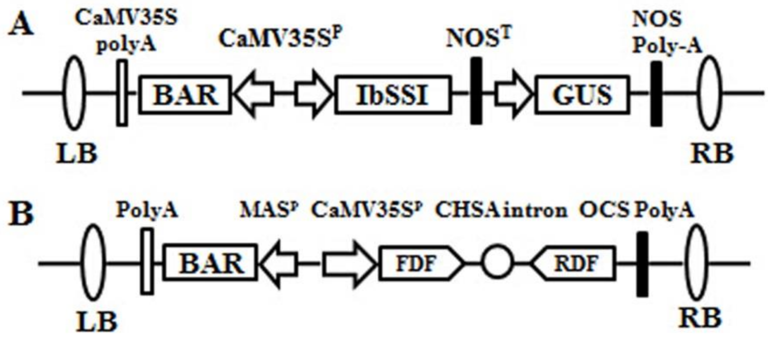

Supplementary Fig. S4. Schematic diagrams of the T-DNA region of the recombinant plasmids pC3301-121-*IbSSI* (A) and pFGC5941-*IbSSI* (B). LB, left border; RB, right border; BAR, Basta resistance protein; CaMV35S^P, cauliflower mosaic virus (CaMV) 35S promoter; NOS^T, nopaline synthase terminator; GUS, *β-glucuronidase* gene; MAS^P, mannopine synthase promoter; CHSA intron, chalcone synthase intron.

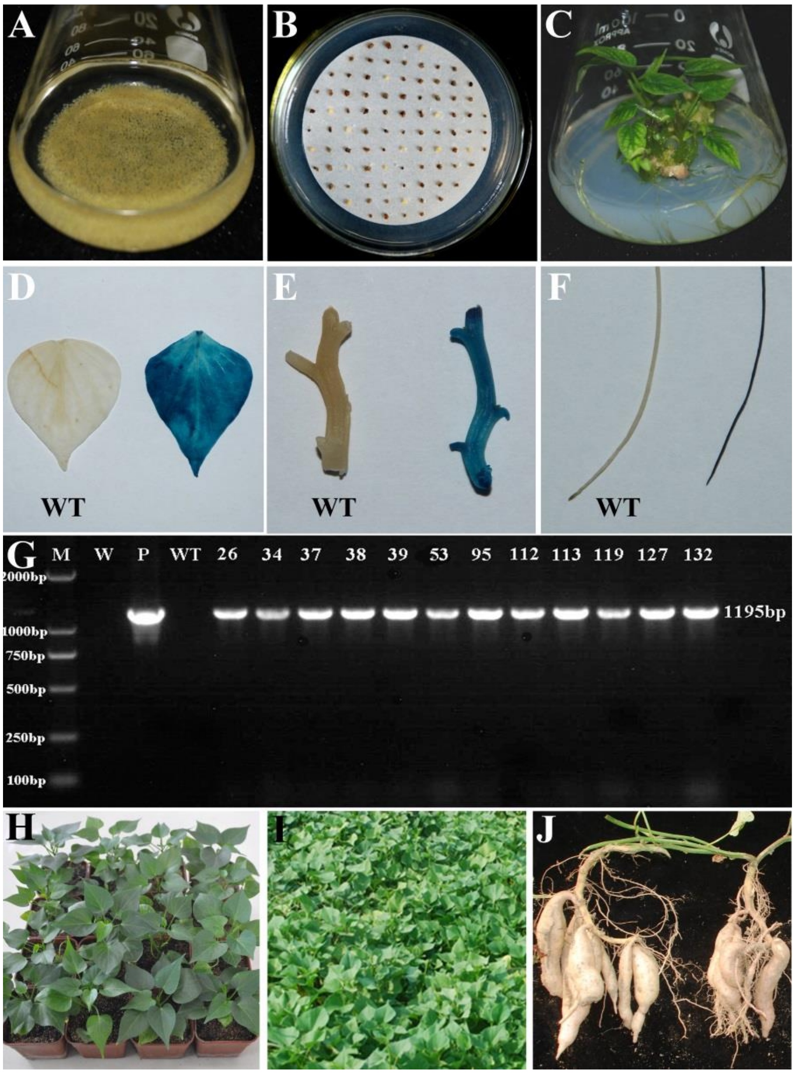

Supplementary Fig. S5. Production of transgenic sweet potato plants overexpressing the *IbSSI* gene. (A) Proliferation of embryogenic suspension cultures of Lizixiang in MS medium containing 2.0 mg L^{-1} 2,4-D. (B) Phosphinothricin (PPT)-resistant calluses (bright yellow) formed after 8 weeks of selection on MS medium containing 2.0 mg L^{-1} 2,4-D, 300 mg L^{-1} cefotaxime sodium and 50 mg L^{-1} PPT. (C) Regeneration of plantlets from PPT-resistant calluses on MS medium containing 1.0 mg L^{-1} ABA and 300 mg L^{-1} cefotaxime sodium. (D-F) The leaves, stems and roots of the transgenic plant showed positive reactions in the GUS assay, whereas no GUS expression was detected in the WT plant. (G) PCR analysis of GUS-positive plants. Lane M: BL2000 DNA marker; Lane W: water as a negative control; Lane P: plasmid pCAMBIA3301-121-*IbSSI* as a positive control; Lane WT: WT as a negative control; Lanes 26-132: GUS-positive plant lines. (H) Transgenic plants grown in the greenhouse. (I) Transgenic plants grown in the field. (J) Storage roots of the transgenic sweet potato.

Supplementary Table S1. Sequences of the primers used in this study

Primer name	Primer sequence (5'-3')
DS-F	GCNTWTGGYGAYAATCAGTT
DS-R	CTTCCWATRAADCCAATCA
5GSP1	CTGCAGTGATGATTGCACCT
5GSP2	TTCACGGTTTCACCAGTGTC
5GSP3	CTTGCCCACGTTGGAAATAC
3GSP1	GCAATCATCACTGCAGATCGCA
3GSP2	AGCCTGCAGTGACGTTCAAGAA
GS-F	ATGGAGGCTCTGTGGGCC
GS-R	CTAGTTCGGCCATCTAACGTATG
GWS1	CAAACACACCACATTTCTGC
GWS2	CACAAAGACGGTCTGCAGCA
ES-F-*EcoR*I	GGAATTCATGGAGGCTCTGTGGGC
ES-R-*Not*I	ATTTGCGGCCGCCTAGTTCGGCCATCTAACGTATG
83S-F-*Spe*I	GACTAGTATGGAGGCTCTGTGGGC
83S-R-*Asc*I	AGGCGCGCCAGTTCGGCCATCTAACGTATG
OS-F-*Xba*I	GCTCTAGAATGGAGGCTCTGTGGGC
OS-R-*Sac*I	CGAGCTCCTAGTTCGGCCATCTAACGTATG
Si-UF-*Xho*I	CCGCTCGAGCTTTCACCGGACCTCTCTGCA
Si-UR-*Swa*I	GCATTTAAATGGAGCCATCCCTCTGTGCTC
Si-DF-*BamH*I	CGGGATCCGGAGCCATCCCTCTGTGCTC
Si-DR-*Xba*I	GCTCTAGACTTTCACCGGACCTCTCTGCA
T35-F	TTGATGTGATATCTCCACTGACG
TS-R	CACGGTTTCACCAGTGTCAAG
int-F	CAACCACAAAAGTATCTATGAGCCT
int-R	TTCACATGTCAGAAACATTCTGATG
QS1-F (*IbSSI*)[a]	GCTGCAGACCGTCTTTGTGC
QS1-R (*IbSSI*)	GAGCCATCCCTCTGTGCTCC

Qactin-F (*IbActin*)	AGCAGCATGAAGATTAAGGTTGTAGCAC
Qactin-R (*IbActin*)	TGGAAAATTAGAAGCACTTCCTGTGAAC
QAGS1-F (*IbAGP-sTL1*)	AGAGAATTGACGGTGATGTTAGCA
QAGS1-R (*IbAGP-sTL1*)	ATGAACGGAGCAGTCCGAAC
QAGS2-F (*IbAGP-sTL2*)	CCAAAAGGAGAACAGTTGAAAGCTA
QAGS2-R (*IbAGP-sTL2*)	CTCCAGGGAACTTTTCTCGAAGTA
QAGL-F (*IbAGP-TLI*)	GAGATATCCCACATCCAACGACTT
QAGL-R (*IbAGP-TLI*)	TAGGGCCAAGTTAGCGTCGTAG
QGB-F (*IbGBSSI*)	TGGCAACTATAACTGCCTCACAC
QGB-R (*IbGBSSI*)	GGCACTGGTTCTCAATTGTAACAT
QS2-F (*IbSSII*)	AGACTGTGGGATCTACTGAAAGGC
QS2-R (*IbSSII*)	GTGAATCCACGTCCAGTGGC
QS3-F (*IbSSIII*)	TCTGTTATCCTGAGGAGGTAAAACC
QS3-R (*IbSSIII*)	CTCCCATGATCAATACATCAGGC
QS4-F (*IbSSIV*)	CTGCTTTCTCATTTCTGTCATCGT
QS4-R (*IbSSIV*)	GCTCAACTTCCACTTGACTCAGAG
QBE1-F (*IbSBEI*)	ATTCTTGGCCTAGACCAAGGG
QBE1-R (*IbSBEI*)	ACAATGCAGCCTTCTTCTTTGTTA
QBE2-F (*IbSBEII*)	AGTCCGCTGTTTGGAGGCTT
QBE2-R (*IbSBEII*)	CCTCAACTGGTTTTGCTTCGTC
QISA-F (*IbIsa1*)	GGAACGAGGTGGTTATCGGTG
QISA-R (*IbIsa1*)	TCTGGGCATAGCAACAGAATTATG
QPUL-F (*IbPUL*)	GCTGCTCGACGATGCCTCT
QPUL-R (*IbPUL*)	CATCCTCAACGTCCACATTCC

[a] Primers used for the qRT-PCR reactions begin with the letter “Q”, and their corresponding genes are shown in parentheses.

Supplementary Table S2. GenBank accession numbers of the plant starch synthases used in the multiple sequence alignment and phylogenetic tree

Species	Gene	GenBank accession number
Ipomoea batatas	*IbSSII*	AF068834
Ipomoea batatas	*IbGBSSI*	AB071604
Solanum tuberosum	*StSSI*	Y10416
Solanum tuberosum	*StSSII*	X87988
Solanum tuberosum	*StSSIII*	X94400
Solanum tuberosum	*StGBSSI*	X58453
Arabidopsis thaliana	*AtSSI*	NP_197818
Arabidopsis thaliana	*AtSSII*	NM_110984
Arabidopsis thaliana	*AtSSIII*	NM_101044
Arabidopsis thaliana	*AtSSIV*	NM_117934
Zea mays	*ZmSSI*	AF036891
Zea mays	*ZmSSIII*	AF023159
Triticum aestivum	*TaSSI*	AF091803
Triticum aestivum	*TaSSIV*	AY044844
Triticum aestivum	*TaGBSSII*	AF109395
Oryza sativa	*OsSSI*	D16202
Oryza sativa	*OsSSII-1*	AF383878
Oryza sativa	*OsSSIII-1*	AF432915
Oryza sativa	*OsSSIV-1*	AY100470
Oryza sativa	*OsGBSSI*	X65183
Oryza sativa	*OsGBSSII*	AY069940

Supplementary Table S3. Enzyme activity assay of IbSSI in *E. coli*

Plasmid	Protein (mg/ml)	Specific activity[a] (unit/mg protein)
pET-28a (no insertion)	8.76	6.07 ± 0.09
pET-28a-*IbSSI*	7.46	29.50 ± 0.95**[b]

[a] One unit of activity is defined as the formation of 1 nmol ADP per min at 30℃.

[b] Data are presented as the mean ± SE (n = 3). ** indicates a significant difference versus the native plasmid sample at P<0.01, based on Student's *t*-test.

Supplementary Table S4. Cis-acting regulatory elements detected in the 5'-promoter region of the *IbSSI* gene

Position	Name	Sequence	Predicted function
-239[a]	G-box	GACGTG	stress-related response
-1159, -1130	ERE	AWTTCAAA	ethylene responsive element
-940	W-box	TTGAC	salicylic acid (SA)-induced for WRKY protein binding site
-893	TAAAG motif	TAAAG	Dof protein binding site
-1558, -239	MYC recognition site	CANNTG	abscisic acid/cold response
-1707	MYB recognition site	CNGTTR	dehydration/water stress
-362	LTRE (low temperature response element)	CCGAAA	low temperature response
-1789	GCC box	GCCGCC	ethylene/jasmonate response
**-379**	**CMSRE-1(Carbohydrate Metabolite Signal Responsive Element 1)**	**TGGACGG**	**sucrose response**
-738	DRE(dehydration-responsive element)	RCCGAC	drought/cold response

-505	(CA)n element	CNAACAC	embryo and endosperm-specific transcription
-1769, -566	HSE(heat shock element)	CCAAT	heat shock response
-20	GT-rich motif	TCTCTCTCT	gene expression enhancer
-841	TGACG motif	TGACG	auxin/salicylic acid response
-249	GATA box	GATA	transcription factor binding site

[a] The first nucleotide "A" in the start codon was designated as +1.
